# Assessment of morbidity due to *Schistosoma japonicum* infection in China

**DOI:** 10.1186/2049-9957-3-6

**Published:** 2014-02-14

**Authors:** Ming-gang Chen

**Affiliations:** 1 National Institute of Parasitic Diseases, Chinese Center for Disease Control and Prevention, Shanghai, People’s Republic of China; 2 WHO Collaborative Center for Malaria, Schistosomiasis and Filariasis, Shanghai, People’s Republic of China

**Keywords:** *Schistosoma japonicum*, Schistosomiasis, Morbidity, China

## Abstract

This paper presents a historical assessment of morbidity due to the *Schistosoma japonicum* infection in China. Due to the socio-economic situation, which did not allow for a control program to be implemented until the early 1950s, morbidity was serious and mortality was high before this. Based on a few investigations and published papers, it can be said that the disease caused millions of deaths, and destroyed numerous families and villages. Since the 1950s, there has been a national control program, intensive control and prevention work has been carried out, and consequently the disease is being controlled. At present, both the prevalence and the morbidity of the disease have been decreasing substantially. The morbidity of the three phases of the disease is outlined in this paper. Comparatively higher morbidity is seen in the acute and advanced phases of the disease. The four major forms of advanced schistosomiasis i.e., ascites, megalosplenia, dwarfism, and colonic tumoroid proliferation, are outlined with their characteristic clinical presentations; their proportions are different during various periods of the national control program. Ectopic schistosomiasis and the relationship between the *S. japonicum* infection and colorectal cancer are also discussed. Post-transmission schistosomiasis is briefly discussed (which can happen even if the disease reaches the criteria of elimination, and the infection and transmission have stopped, but yet it still develops). The problem of mammalian reservoir hosts of *S. japonicum* makes the epidemiology and control of schistosomiasis in China even more complicated and arduous, and the control progress in animal reservoirs is briefly presented.

## Multilingual abstract

Please see Additional file 1 for translations of the abstract into six official working languages of the United Nations.

## Review

Schistosomiasis in China is mainly caused by the infection of *Schistosoma japonicum*. Schistosomiasis japonica has a long history for more than 2100 years evidenced by the finding of the eggs in the liver of an ancient corpse. The first clinical case was discovered in 1905 confirmed by typical S. japonicum eggs found in a patient’s stool. The disease caused millions of death in the last century. Both the morbidity and mortality were considerably high. Scientific records on the morbidity of the disease were few before the founding of the People’s Republic of China in 1949. After 1949, facing the serious situation Chinese Government has made great efforts in implementing a national control program and, as a result, both the prevalence and morbidity have decreased significantly and in large areas are now free from S. japonicum infection. Historical records are appalling that is unique in the world both in *S. japonicum*- and other *Schistosoma*- prevalent countries. Numerous deceased persons, broken families, and destroyed villages could be found in the endemic areas. The morbidity was serious even in the early phase of the national control program period. The epidemic of schistosomiasis on marshlands in Gaoyou County in 1950 caused 4,019 out of 5,257 villagers acquiring acute infection and 1,335 (25.4% of the local population) died that year from schistosomiasis. The highest record in intensity of an artificial infection test in sentinel mice in China was seen in Guichi County with 1,161 schistosomes in a single deceased mouse. The prevalence rate of cattle and buffaloes in the area was 100%. A few farms raising cattle, sheep or goats were closed as most, or all, of the domestic mammals died in the 1950s and 1960s in Hunan, Jiangxi, and Anhui provinces, mainly due to heavy infection of the parasites. Clinical disease is mainly seen in the acute and advanced phases and a number of photos are presenting showing historical morbidity. Nowadays the number of acute and advanced schistosomiasis is much less than it was before. Animal reservoir is a problem for *S. japonicum* in China which increases the complicity in the epidemiology and control of the disease both to humans and animal husbandry. However, the prevalence in domestic animals is under effective control.

### Introduction

Of the five major schistosomes that infect man**—***Schistosoma japonicum*, *S. mansoni*, *S. haematobium*, *S. intercalatum*, and *S. mekongi***—***S. japonicum* is considered to cause the most serious disease. This may be due to the fact that *S. japonicum* female worms have a much higher egg output and that the eggs are laid in large aggregates that induce intensive tissue reactions in host organs. In addition, the life span of the adult worm is probably the longest [1,2]. Schistosomiasis in China is mainly caused by the infection of *S. japonicum*. A zoophilic strain of *S. japonicum* that infects only domestic animals (pigs, dogs, cattle, goats, etc.) and can experimentally infect mice, but has not been shown to infect man, occurs in Taiwan, China [3-5]. Scientists named the parasite as the Taiwan strain of *S. japonicum*[6]. Early in this century, a few human clinical cases of a new species of *Schistosoma*, i.e., *Schistosoma nanjingi*, were reported with the discovery of eggs both in the feces and the urine of human hosts, and were restricted to limited endemic areas close to Nanjing City, Jiangsu Province [7]. However, information on the morbidity caused by the infection with this new species is very limited. This paper deals only with the major species, *S. japonicum*, presenting a historical assessment on morbidity in humans and, to some extent, in domestic animals.

### Historical records before 1949

**Figure 1:**
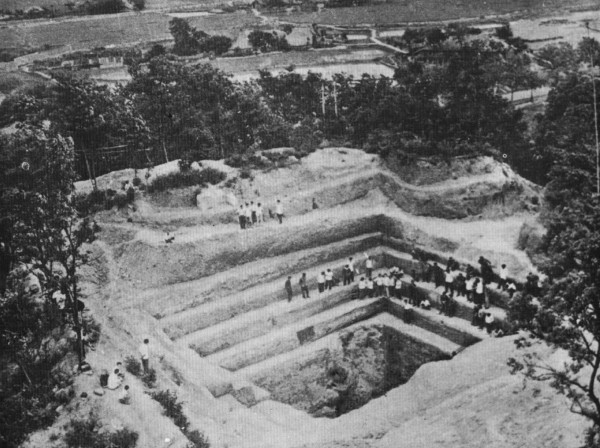
**Excavation site of an ancient female corpse with schistosomiasis in Changsha City, Hunan Province, in 1971 **[8]**.**

**Figure 2:**
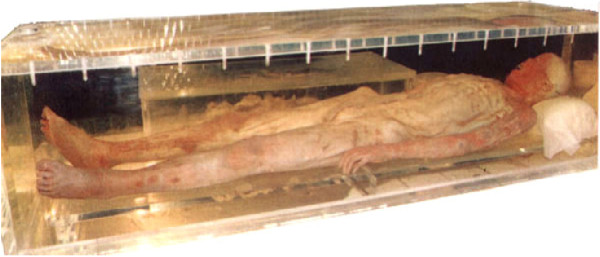
**Mummy prepared from the corpse of the excavation **[8]**.**

**Figure 3:**
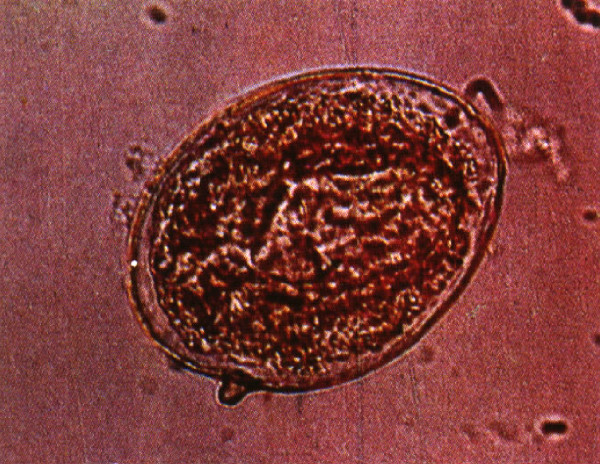
**An egg of *Schistosoma japonicum *** **isolated from the liver of the corpse showing a miracidium inside and a small exterior spine (Photo provided by Prof. Li Yunhe).**

**Figure 4:**
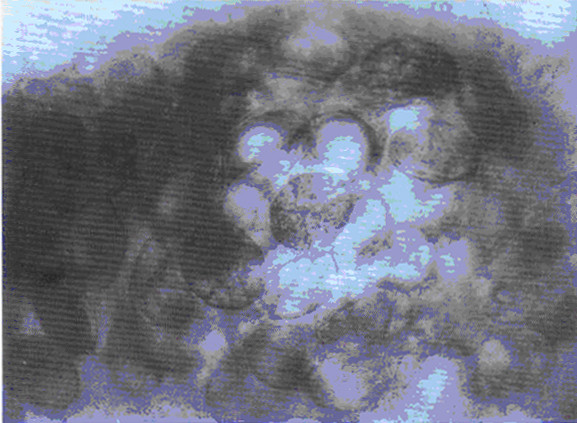
Schistosome eggs in clumps from the rectum mucosa of the corpse (Photo provided by Prof. Li Yunhe.).

The eggs of *S. japonicum* identified in two ancient corpses from the Hunan and Hubei provinces excavated in 1971 and 1975, respectively, have shown that the prevalence of schistosomiasis in China has a history dating more than 2,100 years [8-10] (see Figures 1, 2, 3, and 4). In old volumes about traditional Chinese medicine, a description of clinical symptoms resembling Katayama fever (acute schistosomiasis) can be traced back to 400 B.C. [11], and symptoms resembling the late stage of schistosomiasis, such as ascites and splenomegaly, can be traced back to as early as 2697 B.C., as evident in the text “Ling-Su”, claimed to be written by Huang-Di (the King) [12,13]. Great suffering and premature death due to schistosomiasis existed for centuries [11]. The first clinical case was discovered in 1905 in a missionary hospital in Changde County, Hunan Province, by Dr. Logan, an American physician, confirmed by typical *S. japonicum* eggs found in the patient’s feces [14]. The patient was an 18-year-old fisherman. His major complaints were bloody diarrhea and loss of working ability. A physical examination showed an underdeveloped young man with a short statue (137 cm in height). In 1924, Dr. Totell, also an American physician and director of the aforementioned missionary hospital, examined 63 residents in two villages in the same county using the direct fecal smear technique (a technique with low sensitivity for the discovery of the *S. japonicum* infection). Eggs were found in 38 of the fecal specimens with a high prevalence of 60.3%, showing that both prevalence and intensity of the infection in the area were very high [15]. Faust and Meleney, professors at the Peking Union Medical College, were the first to make a survey on the infection in the Jiangsu, Zhejiang, and Guangdong provinces, and to discover the molluscan host of the parasite in China. They published a monograph “Studies on schistosomiasis japonica” in 1924, which was the first systemic report related to the prevalence and morbidity of the *S. japonicum* infection published in the country [16]. Clinical cases and valuable observations on the morphology and lifecycle of the causative agent and pathological anatomy of the disease were provided.

A few papers related to the prevalence and morbidity of the infection and control of the disease in different provinces have been published in Chinese and foreign medical journals by Chinese and foreign scientists prior to 1949 [17-20]. Hsu and Wu summed up the distribution of schistosomiasis in 1941 based on a literature review and their own experiences in the field [21]. Mao and Shao wrote a detailed review on the epidemiology of schistosomiasis japonica in China that included geographical distribution, zoological distribution, and incidence [11]. The disease was claimed to be endemic in 138 counties in 11 provinces, i.e., Jiangsu (including suburbs of Shanghai), Zhejiang, Anhui, Jiangxi, Hubei, Hunan, Fujian, Guangdong, Guangxi, Sichuan, and Yunnan. Based on two review papers, it was estimated that several dozen million persons were infected with *S. japonicum*[11,21]. Several species of mammalian hosts were found to harbor the infection including dogs, cats, rats, cattle, buffaloes, sheep, and goats [11,22-24].

Three species of schistosomes, i.e., *S. japonicum*, *S. mansoni*, and *S. haematobium*, account for the majority of the human infection. Schistosomiasis, as a special disease, was evident in Katayama, Japan as early as 1847, however, *S. japonicum* was discovered by Katsurada in 1904 in domestic animals [25,26]. The first human *S. japonicum* infection case was reported in Japan by Fujinami who found adult worms in the portal vein of the liver upon necropsy in 1907. In contrast, although Bilharz identified the cause of African schistosomiasis in 1852, the picture of transmission was complicated as two species (*S. mansoni*, *S. haematobium*) of worms were involved. The lifecycles of the two species of *Schistosoma* remained a mystery for more than 60 years until 1915–1918 [26-28].

Morbidity due to the *S. japonicum* infection in China was very high according to the reports written prior to 1949, and field investigations in the 1950s and early 1960s were supplemented with recalls from local people and governments. Before the 1950s, numerous deceased persons, broken families, and destroyed villages could be found in the disease endemic areas in the southern part of China. Before 1949 and in the early phase of the national control program in the 1950s, schistosomiasis endemic areas were called by horrible names, such as “no man’s village” or “village without villagers” (in cases where most of the villagers died and others fled their homes to seek safety elsewhere in fear of some ghost remaining in the village who was diminishing the strength of their lives), “widows’ village” (when most of the men in the endemic villages died because of their more frequent water contact than women), “big belly village” (with many of the villagers suffering from hepatosplenomegaly and/or ascites), and “dwarf village” (with many of the villagers being short in stature and sexually underdeveloped due to a schistosome infection during childhood) [29-31]. The situation was serious in terms of the morbidity and mortality of the disease that China was facing after the founding of the People’s Republic in 1949, not only from the prevalent status of the time, but also from the appalling historical records caused by the prevalence of the disease.

According to retrospective surveys in the 1950s, in 34 counties in the Jiangxi Province endemic for schistosomiasis, a total of 1,362 villages were destroyed, 26,000 families died, and 310,000 residents departed in a period of 40 years before 1950. The major cause of death was estimated to be due to schistosomiasis, which accounted for approximately 90% of the deaths. One village, Gentou, in Fenchang County – once a large village with about 1,000 households by the end of the 19th century – had decreased to only two households in 1954 [31]. In the Shangyanpan village, Yushan County, with a population of over 500 30 years before 1949, only 144 remained, of whom 115 were schistosomiasis victims, and there were widows almost in every household. Many families had three generations of women widowed in succession. Thus, Shangyanpan was known as the “Village of Widows” [32]. Such villages could also be found in other provinces in the endemic areas. In Jiaxing County, Zhejiang Province, during a period of less than 30 years prior to 1949, about 30,000 persons died from schistosomiasis and 286 villages were destroyed. As most of the labor force was sick due to the infection, 1,860 hectares of farmland were wasted [33]. In the 1940s, 17,000 persons died from the “big belly disease” (patients with hepatosplenomegaly and/or ascites) in Jiashan County, Zhejiang, mainly from schistosomiasis [32]. In Yangxin County, Hubei Province, one of the 10 most heavily endemic counties in China of the last century, around 700 villages were destroyed during the 20 years preceding 1949 and resulted in 15,000 hectares of farmland being deserted [31]. In Tzeshih village, Kangning County, Hubei Province, once well known for its rich yield of rice, the disease levied such a heavy toll on the life and health of its inhabitants that 800 hectares of fertile land were overgrown with weeds; and scourged by famine, people had to eat weeds and husks, or beg elsewhere [32]. In Rentun village, Qingpu County, Shanghai Municipality, more than 500 persons died from schistosomiasis before 1949 with 97 whole families dying out, while in the remaining 28 households, only one person remained alive. A baby’s crying had not been heard for eight consecutive years in the fourth decade of the 20th century [34]. In Nianzexia village, Guichi County, Anhui Province, all the family members died in 119 out of 120 households, and only four persons in one family were still alive in the early 1950s. Among them, three were patients with schistosomiasis, and the other one was a barber with a much smaller chance of coming into contact with schistosome-infested water [31]. In desperation, some victims even sought salvation in death. Shortly before 1949, in Fenghuangtai village, Xiangyin County, Hunan Province, 11 people ended the nightmare of their existence by suicide [32]. By the first half of the 20th century, quite a few patients committed suicide in other areas by either hanging themselves, drowning in rivers or wells, or knife cutting at their inflated abdomen with ascites, which is a symptom of the long-term, serious suffering of the disease [31].

Such horrible examples can be seen in many counties in the endemic areas in China and have been recorded in the county annals in the endemic provinces. Why was the damage of schistosomiasis so serious in China before the 1950s? Obviously, the morbidity of the disease was high, and control work was hardly carried out due to the socio-economic situation. Even children from richer families, such as landlords in the countryside, acquired the infection as their families could not afford treatment with the anti-schistosome drug**—**then tartar emetic. The cost of treatment was so high that only several rich families could afford treatment for just one child. Families with more than one infected child drew lots to determine which child to treat, and an equal sum of money were paid by each family with one child patient. The other children were not treated and could only await death [35]. Importantly, what also added to the seriousness of the situation was that apart from humans, many species of mammals can be infected with *S. japonicum* and serve as reservoir hosts, i.e., are important sources of the infection [2,28]. By the late 1940s, only a few surveys were done by scientists in China about animal reservoirs [12,22,34].

People in the endemic areas who had long been suffering from the disease not knowing its cause, were, naturally, extremely superstitious. Once they became infected with the parasite, they either attributed it to the unfavorable “wind and water” of their ancestral tombs, or suspected the presence of evil spirits in the lakes [31]. The people bitterly lamented their fate in verse: “After father’s death, nobody carries him; after son’s death, nobody buries him. Foxes and rabbits the village roam; weeds only fill the rooms” [32]. “Died are thousands of villages, crying and singing are only ghosts.” The situation was vividly described in the famous poem, *Farewell, God of Plague*, written in 1958 by Mao Zedong, late chairman of the Chinese Central Government. Mao wrote the poem knowing about the epidemic situation of schistosomiasis before the 1950s.

### The national control program and its progress

Little can be said about the control program before the 1950s, as it did not exist. In recognition of the devastating effects of schistosomiasis on rural areas and people’s health, a national control program was established in 1955, and large-scale, intensive control work has been carried out in the past six decades. Several nationwide surveys have been executed in the past five decades and the distribution of the infection has delineated. Both the infected subjects and *Oncomelania* snail intermediate hosts have been found in 454 counties along and in the south of the Yangzi River in the southern part of China, which includes 10 provinces, the municipality of Shanghai, and the autonomous region of Guangxi [36]. By the end of 2011, the criteria for elimination of schistosomiasis [37] was reached in 274 counties in Guangdong, Shanghai, Fujian, Guangxi, and Zhejiang provinces, municipality and autonomous regions, and**—**in the remaining counties still endemic for the infection with different endemicities**—**the situation has changed significantly. The total number of infected persons in the endemic areas was estimated to be around 10 million in the 1950s [31]. By 1989, 1995, and 2004, three large-scale, well-organized nationwide sampling surveys on the epidemic status of schistosomiasis were carried out. The infected persons were estimated to be as about 1,522,100 in the 1989 survey [38], 865,084 in the 1995 survey, a 91.4% decrease [39], and 726,111 in the 2004 survey, a decrease of 92.7% [40], as compared with the data from the 1950s. According to the case reporting system for infectious diseases collected by the Ministry of Health, the number of infected subjects was calculated to be 286,836 in 2011, a 97.1% decrease [36]. The number of acute cases and advanced cases, sensitive indices for morbidity estimation of schistosomiasis, fluctuated to around 10,000 (from several thousand to 14,000) yearly and was estimated to be one million, respectively, in the 1950s [31]. The number of advanced cases was calculated to be 55,961 in 1995 [39]. The number of advanced cases was 30,028 in 2011, a 97% decrease compared with the figure in the 1950s, while the number of acute cases was only three [36]. The number of infected bovine, including yellow cattle and water buffaloes, was estimated to be around 500,000 in the 1950s [31]. The number of infected bovine was calculated to be 100,251 in 1995 with a positive rate of 9.06%, estimated by conducting a fecal examination for the eggs of *S. japonicum*[39]. In 2011, the eggs of *S. japonicum* were found in 5,146 bovine in the endemic areas via fecal examination or rectal snips (a more sensitive technique for the detection of the infection) among 753,782 (53.4% of the total number of 1,410,936 bovine in the areas still endemic for schistosomiasis) examined with a positive rate of 0.68% [36]. All in all, achievements in schistosomiasis control in China have reduced the human infection rate by more than 97% as compared to the peak estimates in the mid-1950s, and the prevalence in bovine had reduced by more than 98% by 2011 [36]. The decline of the infection and morbidity of *S. japonicum* was significant but gradual as a whole with some fluctuations in the past six decades [34]. The total area of the *Oncomelania* snail habitats in the 1950s and 1960s was about 1.4 million hectares, and 372,664 hectares in 2011 [36], a decrease of about 73%.

Since the national control program started, the prevalence and morbidity of schistosomiasis in China has been decreasing. Let’s take Guichi County, one of the 10 most heavily endemic counties in China as an example. The endemicity and morbidity of schistosomiasis in the county was serious in the 1950s and 1960s. Fecal examination showed that the prevalence of residents in the county town around the Southeast Lake was as high as 70%, the majority of the infected subjects were symptomatic, and over 70% were showing signs of hepatomegaly and/or splenomegaly in 1956. The infection rate of *S. japonicum* in sentinel mice was 100% in repeated testing with hundreds of mice. The intensity of the infection in mice was 449 worms per mouse four week after a standardized technique**—**one-hour water contact per day for 10 consecutive days. Up until now, the highest record of artificial infection intensity in sentinel mice in China was seen in the area with 1,161 schistosomes in a single deceased mouse due to the infection. The prevalence rate of cattle and buffaloes in the area was almost 100%. In the 1950s, outbreak of acute schistosomiasis was seen almost every year, and there were many advanced cases [41]. After intensive control work in the 1980s, the endemic situation greatly improved. After environmental modification to make the swamp and lake into cultivable land, as well as other intensive, combined prevention and treatment approaches, the county town and its surrounding area were free from schistosomiasis as shown by human and animal examinations, the snail survey, as well as sentinel mice testing [41].

### Morbidity of the disease after 1949

The assessment of morbidity due to the *S. japonicum* infection has mainly been based on prevalence, infection intensity, clinical presentations, and mortality. Recently, the quality of life and disability weight due to schistosomiasis has been taken into account as a quantitative estimate on the disease burden [42-44].

Along with the sharp decrease of the prevalence, as well as the intensity of the infection, morbidity of the disease has significantly declined in the past six decades. Clinical manifestations have changed considerably compared with those in the early stage of the national control program. Thousands of scientific papers related to morbidity of schistosomiasis japonica in China have been published in the country’s biomedical journals, especially professional journals, such as the *Chinese Journal of Schistosomiasis Control,* the *Chinese Journal of Parasitology and Parasitic Diseases*, etc., however, most of them are in Chinese.

Clinically, there are three phases for schistosomiasis in China: acute phase, chronic phase, and advanced phase. The morbidity and mortality were comparatively high in the advanced cases, and morbidity of acute schistosomiasis was very high with a considerably higher mortality before Praziquantel was available for treatment. Ectopic schistosomiasis is another type of the disease that may be seen in different phases of schistosomiasis.

### Acute schistosomiasis

**Figure 5:**
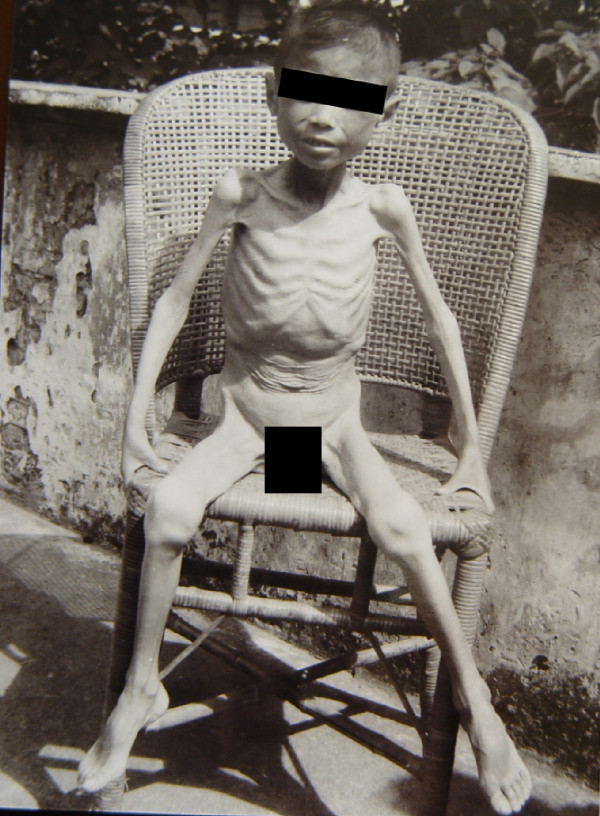
**Terminal phase of a 12-year-old boy suffering from acute schistosomiasis in Changshu County in 1965, a week before his death **[2]** (Author’s collection).**

The clinical manifestation of acute infection with *S. japonicum* is outstanding, and it was considered to be more serious than that of the infection with *S. mansoni*, *S. haematobium*, and *S. intercalatum*[1,2,28]. Clinical presentations of the acute disease include fever, rigor, sweating, headache, general muscular pain, gastrointestinal disturbances, enlargement and tenderness of the liver, and eosinophilia. Serious patients are usually very sick with high-grade fever, and if not treated in time, the subjects may die. Figure 5 shows a shocking image of an acute case at the terminal stage of the disease, taken in 1965. Although the patient was diagnosed with the acute disease in time and treated with two not very effective anti-schistosome chemicals, furapromidum and hexachloroparaxylol (Hetol), in a special hospital for schistosomiasis by experts, his life could not be salvaged as Praziquantel was not yet available. The frequency of the acute infection is presently much lower than it was in the 1950s and 1960s. The number of persons identified as having acute schistosomiasis was less than 100 per year for three consecutive years by 2011, according to the infectious disease reporting system in China [36].

The following are several examples of the outbreak of the acute disease after 1949.

In 1949 and 1950, 33,891 soldiers and cadres of the Chinese Liberation Army were infected with *S. japonicum,* most of them with the acute infection, and lost their capacity to participate in military exercises and to swim in the water in the endemic areas of the Yangtze River Delta in the provinces of Jiangsu and Zhejiang, and suburbs of Shanghai City. The fighting ability of three divisions of the army force was thus lost [31]. During a flood season in 1950, in Xinmin township, Gaoyou County, Jiangsu Province, 4,019 out of 5,257 villagers acquired the acute disease after coming into contact with schistosome cercaria-infested water, used for the collection of wheat, reed leaves, and vegetable oil seeds or fishing on marshlands, and 1,335 (25.4% of the local population) died that year. The mortality rate due to the acute infection of *S. japonicum* was extremely high probably due to the high density of schistosome cercaria on the marshlands. Hundreds of corpses, some of them even without coffins, were disposed on the dykes around the marshlands, forming a distance of 9 km long [31,45]. This big event, in combination with a high incidence of outbreaks of schistosomiasis in the armies during 1949–1950 in the Yangtze River Delta, and other epidemics of the disease elsewhere in the early 1950s, propelled the Chinese Government to pay greater attention to schistosomiasis and its control work.

In 1962, in Yuanjiang County, Hunan Province, 1,762 persons got the acute infection and 61 died [31]. In the Hubei Province, where the extensive marshlands are highly endemic, 2,984 persons in 24 counties were reported to acquire acute infections in 1983. In a single village, 95 persons acquired the acute infection among 1,034 persons exposed to the infested water [46]. In 1989, in Wuhan City areas and its suburb along the Yangzi River, thousands of city residents and students acquired the acute disease by swimming in the water during the hot summer [34]. As local hospitals were full of patients, children stopped attending school and classrooms were used as temporary wards for the treatment of the disease. Also in 1989, in the suburb of Nanjing City, an outbreak of acute schistosomiasis happened in 364 subjects mainly because they collected reed leaves in the infested water [47].

### Chronic schistosomiasis

*S. japonicum* infected subjects may be symptomless. People in endemic areas with comparatively smaller chances of contact with infested water, and those with the infection and incomplete treatment, may enter the chronic phase. Most subjects with the infection in the endemic areas are chronic cases. Some are symptomless and some are slightly symptomatic, and the impact to their working ability is usually not significant. If not treated in time, the disease may become advanced causing higher morbidity, but some patients may be latent all their life. Conducting autopsies in deceased patients who had diseases other than schistosomiasis, schistosome eggs and egg granulomas were found in the tissues of a number of corpses without histories of identification of schistosomiasis and anti-schistosome treatment during their lifetimes [28].

### Advanced schistosomiasis

Most of the mortality of schistosomiasis is seen in the advanced stage. Four major forms of advanced schistosomiasis have been classified in China, i.e., ascites, megalosplenia, dwarfism, and colonic tumoroid proliferation [10,28,48].

**Figure 6:**
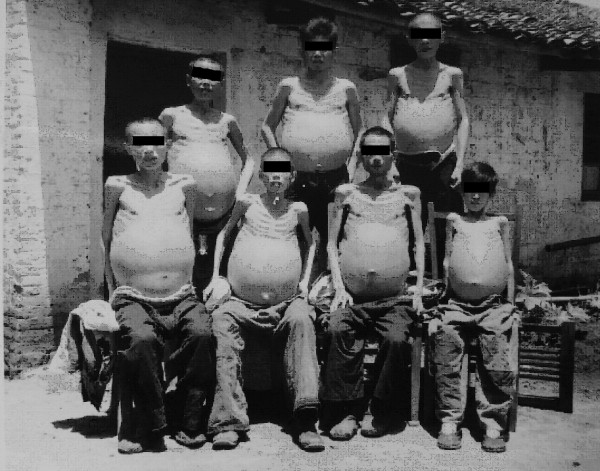
Collective photo of male advanced cases with ascites in the Guizi County Anti-schistosomiasis Hospital in 1959 (Author’s collection).

**Figure 7:**
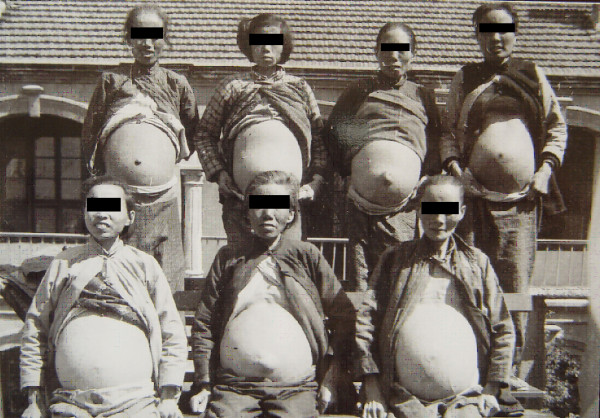
Collective photo of female advanced cases of schistosomiasis in the Changshu County Hospital in 1965 (Author’s collection).

**Figure 8:**
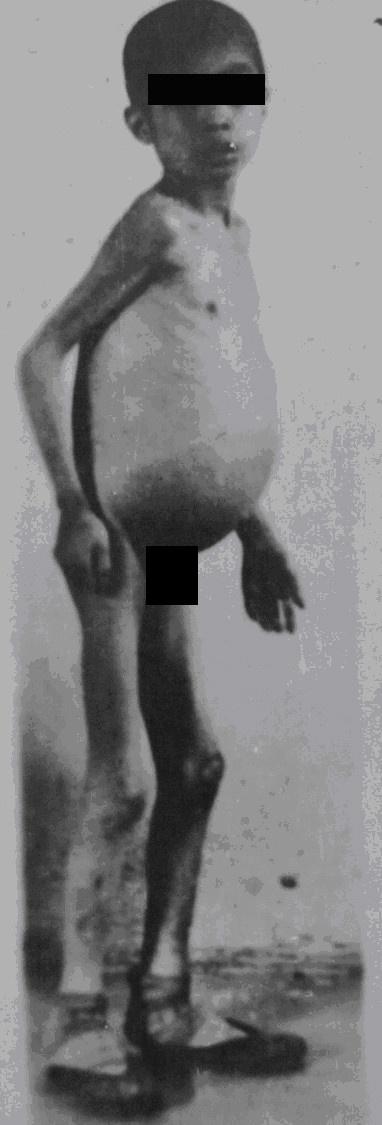
An advanced case with ascites in Gaoyou County, Jiangsu Province in 1958 (Author’s collection).

**Figure 9:**
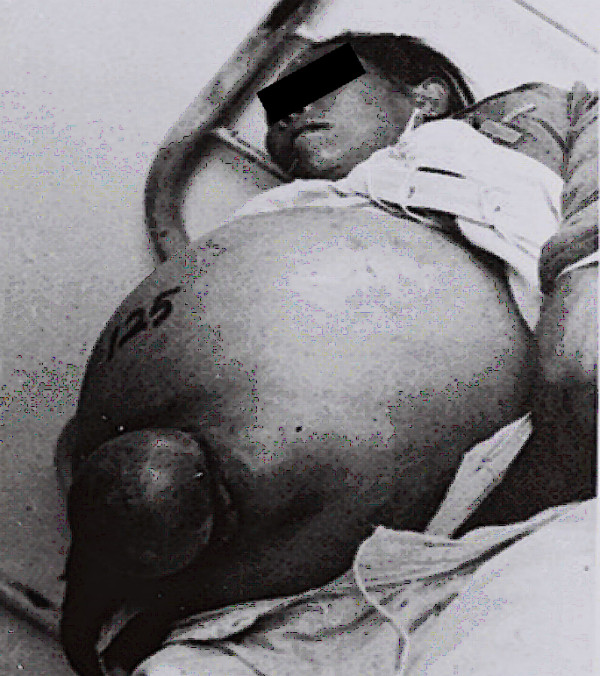
An advanced case with a huge amount of ascites (abdominal circle 125 cm) and umbilicus hernia in Changshu County in 1965 (Author’s collection).

Patients with ascites were considered to be the most serious among all forms of advanced schistosomiasis. Ascetic patients due to schistosomiasis were commonly seen in the endemic areas. In the 1950s and 1960s, they consisted of about one third of the advanced disease, and the number of the subjects was estimated to be as high as several hundred thousands in whole country [34]. In the special hospitals for schistosomiasis with 50 to 100 beds in heavily endemic areas, dozens of ascetic patients could be found, as shown by Figures 6 and 7, which were taken in small hospitals in 1959 in the Guichi County, Anhui Province, and in 1965 in Changshu County, Jiangsu Province, respectively. The collective photos of advanced cases with ascites could only be taken in the earlier phase of the national control program, i.e., in the 1950s until the 1970s, and, thanks to the intensive control program, after 1980, the number of ascetic patients significantly decreased. Individual photos of advanced schistosomiasis are shown in Figures 8 and 9. The volume of ascites in one advanced case was documented as large as 20 liters, about one third of his body weight, as assessed by the technique of repeated abdominal paracentesis and injection with methylene blue. Along with the progress of the control effect, the number of ascetic patients due to schistosomiasis has considerably decreased in recent years [36,49].

**Figure 10:**
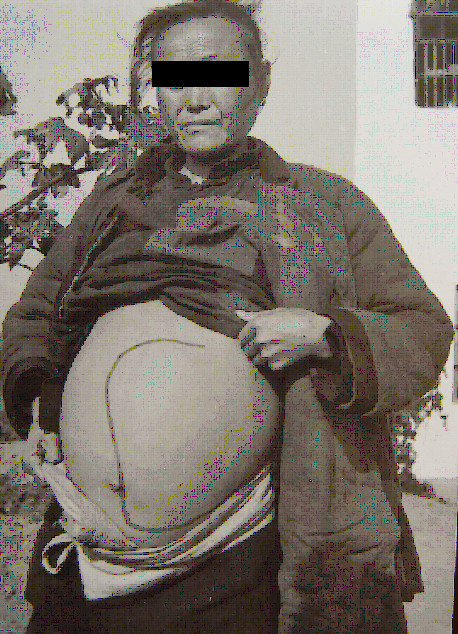
An advanced case with a huge spleen (the size indicated with a black marker) in 1960 in Guichi County (Author’s collection).

Megalosplenia is another form of advanced schistosomiasis quite commonly seen in the 1950s and 1960s. Persons usually have a very large spleen and hypersplenism. The enlarged spleen may be palpated across the level of umbilicus or abdominal midline, i.e., ≧3 in Hackett size, as shown in Figure 10. The enlarged spleen usually weighs 1,000 g to 2,000 g at autopsy or upon splenectomy, with the largest one recorded being over 4,000 g [2,31].

Between 1954 and 1977, splenectomy was done to a total of 5,151 patients with megalosplenia and/or hypersplenism due to schistosomiasis in one county, i.e., Qingpu County, Shanghai Municipality, which had very high prevalence and morbidity of schistosomiasis and a total population of about 400,000 [48]. In doing this, a number of surgical teams from big hospitals of Shanghai Medical Colleges and of the Second Military Medical University were dispatched to the endemic fields by the Health Bureau of the Shanghai Municipality Government.

**Figure 11:**
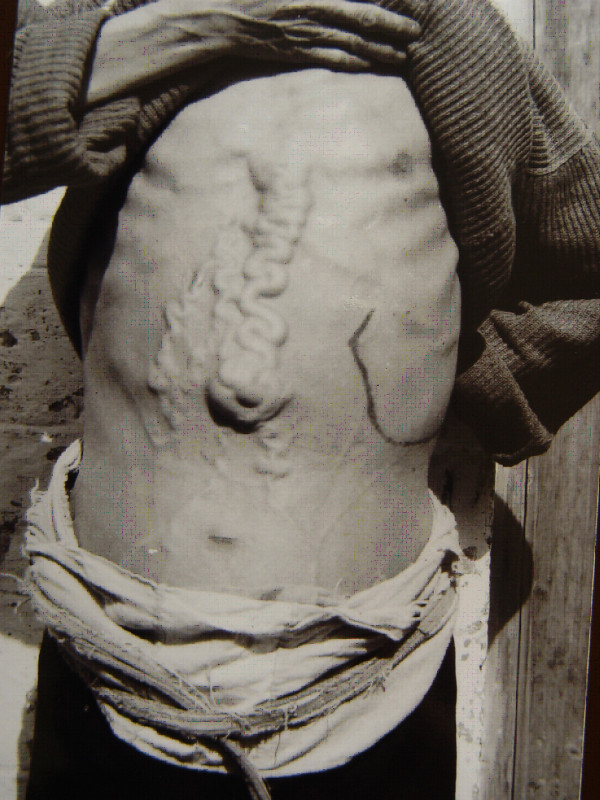
**Dilatation of abdominal collateral veins due to portal hypertension in a patient with advanced schistosomiasis.** The enlarged spleen was marked with black ink. (Author’s collection).

Advanced schistosomiasis with ascites and/or megalosplenia is usually associated with abdominal collateral vein dilation (see Figure 11) and oesophagogastric varices. The rupture of varices occurs mostly at the fundus of the stomach and, secondarily, in the lower third of the oesophagus. Haematemesis and melaena are frequent, and upper gastrointestinal bleeding is the most important cause of death (above 50%) in advanced schistosomiasis. The second important cause of death is hepatic failure with or without going into a hepatic coma [48].

**Figure 12:**
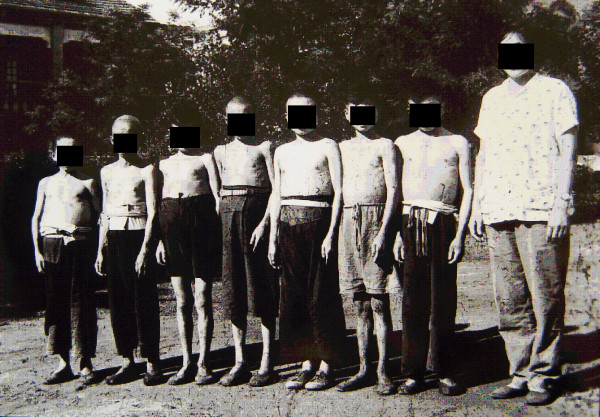
**Seven schistosomiasis dwarfs in Guichi County Anti-schistosomiasis Station standing together, ranging in ages between 16 and 35 years, and all of short stature (shorter than 140 cm in height); the person on the far right was a nurse in the hospital in 1959.** (Author’s collection).

**Figure 13:**
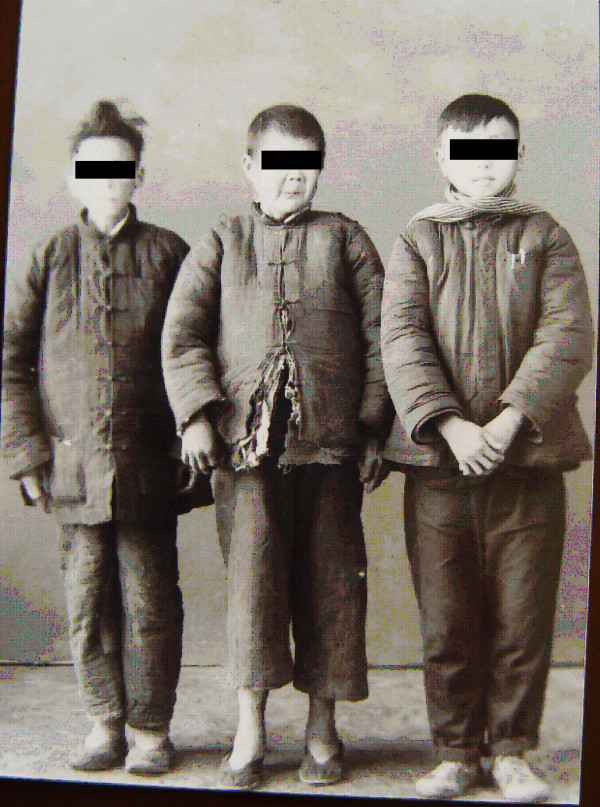
**Persons of almost the same stature (around 140 cm in height) in 1960: the one on the right was 36 years old, the one in the middle was a 45-year old man (both were schistosomiasis dwarfs with older facies), and the one on the left was a 14-year-old student in Susong County, Anhui Province.** (Author’s collection).

**Figure 14:**
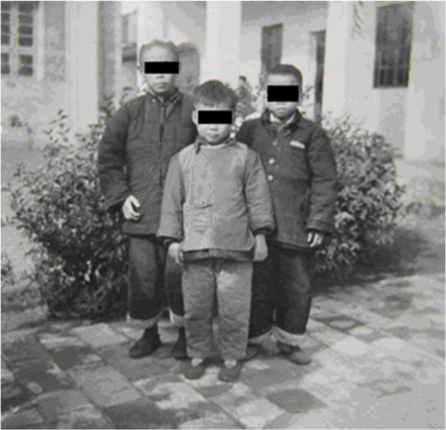
**Three persons standing together with similar statures in 1959: the one on the right was 30 years old, the one on the left was a 20-year-old schistosomiasis dwarf in Guichi County Anti-schistosomiasis Station, and in the middle was a five-year-old kindergarten child, the son of a head nurse of the hospital.** (Author’s collection).

As one of the four major forms of advanced schistosomiasis, schistosomiasis dwarfism was quite common in endemic areas in China in the 1950s and 1960s. Nowadays, it is very rarely seen. These individuals acquire infection, usually repeated and heavy, during childhood. Their physical growth and sexual development is retarded. Lack of growth acceleration during puberty, a short stature with an older facies, a lack of secondary sexual characteristics, and underdeveloped sexual organs are common characteristics. Loss of libido in adults, impotence in men, and infertility and amenorrhoea in women are common in cases with advanced schistosomiasis. Schistosomiasis dwarfs photographed in 1959 and 1960 in two counties of the Anhui Province are shown in Figures 12, 13 and 14. In the 1950s, schistosomiasis dwarfs accounted for 4% of the whole population in the Rentun village, Qingpu County, Shanghai [50].

**Figure 15:**
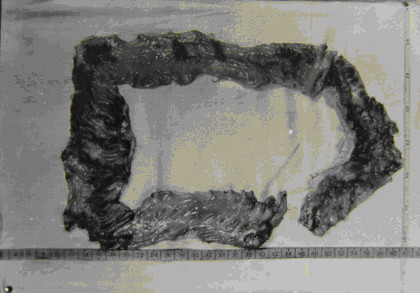
Diffuse involvement and multiple lesions of the large intestine in a schistosomiasis patient during autopsy in 1964 (Author’s collection).

Before the 1980s, the colonic tumoroid proliferation form of the disease, granulomatous disease of the large intestine secondary to schistosome infection, was not uncommon among advanced schistosomiasis, especially in the Jiangsu Province, with 229 cases reported [51]. It has been a rarity in the past two decades, usually comprising less than 1% of the total advanced cases. The patients were usually able to work until the disease became well advanced or complications appeared. Diffuse involvement and multiple lesions of the large intestine were seen in the cases although the disease was mainly localized in the rectum and sigmoid colon (see Figure 15). The complications of colonic obstruction and perforation or intestinal hemorrhage were reported. Based on tissue egg count after digestion done in 10 autopsy cases with advanced schistosomiasis egg concentration in the intestinal tissue, it was the highest in the rectum, then (in descending order) the sigmoid, descending, transverse, and ascending colon, while the small intestine was only slightly affected [52]. This is probably the reason that colonic tumoriod proliferation is comparatively common in advanced schistosomiasis.

### Ectopic schistosomiasis

The egg granuloma lesions of *S. japonicum* and in rare case, adult worms, may be found outside the portal venous system causing ectopic schistosomiasis. Apart from the organs of the portal venous system, almost all organs and tissues in a host’s body can be involved, such as the skin, subcutaneous tissue, lymphonodes, muscles, conjunctiva, tongue, brain, spinal cord, sympathetic nervous system, thyroid, breast, lung, cardiac muscle, pericardium, esophagus, ascetic fluid, kidney, ureter, urethra, bladder, adrenal cortex, inguinal hernia sac, reproductive organs of both sexes etc., and among them, ectopic clinical symptoms and lesions have more frequently been reported in patients with cerebrospinal and pulmonary schistosomiasis [28,53-56].

### Cerebrospinal schistosomiasis

#### Cerebral schistosomiasis

The *S. japonicum* infection mainly affects the brain, with spinal cord involvement rarely reported. No population-based survey is available for the assessment of the prevalence of cerebral involvement due to *S. japonicum*. Among adult hospital patients in China in the 1950s, the prevalence of cerebral schistosomiasis among all patients with schistosomiasis ranged between 1.7% and 4.3% [29,57]. In the early stage of the national control program, i.e., in the 1950s and 1960s, cases with cerebral schistosomiasis were mainly seen in the acute phase of the infection [1,57]. However, during the past two decades, due to the control efforts, acute schistosomiasis has gradually been decreasing and along with it, cerebral schistosomiasis has also been significantly decreasing and is mainly, or almost exclusively, seen in the chronic phase and rarely in the advanced disease [1,28,48].

Clinical manifestations of cerebral schistosomiasis differ considerably between the cases in the acute phase and the chronic phase of the infection. In the former, the usual presentation is meningoencephalitis with or without localized granulomatous findings, but with the clinical symptoms of fever, headache, vomiting, dysphrasia, hemiparesis, blurred vision, disturbance of consciousness, and eosinophilia [28,57,58]. Epileptic seizures usually with granulomatous lesions in the brain are the main clinical manifestations of the cerebral disease in the chronic phase. Symptoms common with brain tumors such as headaches, disturbance of speech, and paralysis often develop. In a large series of cerebral schistosomiasis cases confirmed by surgical therapy and histopathological studies during the period from 1992 to 2012 in a hospital in Jingzhou City, Hubei Province (one of the most heavily endemic counties (cities) in China up until now), it was shown that 42 patients had granulomatous lesions confirmed by magnetic resonance imaging and computed tomography, serological tests, and cerebrospinal fluid tests for anti-schistosome antibodies and pathological examination of the surgical specimens. Many more cases diagnosed clinically as cerebral schistosomiasis and effectively treated in the internal medicine department of the hospital with Praziquantel and corticosteroids were not included. Total resection of the granuloma for those with a single granulomatous lesion in the so-called “non-functional areas” (actually unimportant functional areas) of the brain was performed in 25 cases including associated decompressive therapy in eight cases with intracranial hypertension. Partial resection of the granuloma (s) and decompressive therapy was performed in 17 patients with their granulomatous lesions in more than two lobes, or in the important functional areas of the brain [59].

The pathological changes in the brain are local reactions in response to the presence of eggs, and, although ectopic parasitism of the adult worms in the cerebral vessels has been suggested, up until now, no worm has ever been found in specimens of the human brain. On the other hand, histological sections from surgical operation and necropsy have shown that all were egg granuloma (s) in the brain tissue [32,57-59].

#### Myelonic schistosomiasis

Unlike the *S. mansoni* infection, in which the central nervous system’s involvement was seen more often in the spinal cord than in the brain, egg granulomas in the spinal cord and adult worms in the subarachnoid veins of the spinal cord were found in human cases [60]; spinal cord involvement in *S. japonicum* infection is very rare. Clinically presenting transverse myelitis in acute *S. japonicum* infection was presumptively diagnosed as myelonic schistosomiasis only in a few cases, and the symptoms were released after etiological treatment with trivalent antimony. Only one case diagnosed as spinal cord schistosomiasis and confirmed with histopathological evidence by the finding of *S. japonicum* egg granuloma in the tissue was reported in the Chinese literature [28].

### Pulmonary schistosomiasis

Clinical pulmonary schistosomiasis was frequently seen (about 30–70%) in the acute phase of the *S. japonicum* infection, especially in the earlier stage of the national control program, i.e., from the 1950s to the 1970s, when the yearly incidence of the acute infection was high [1,28,31]. Radiological appearances of the chest were well documented in the earlier Chinese literature. On X-ray film, diffuse, mottled opacities, or military shadows, poorly or well defined according to the stage of the acute infection, were shown. Hazy shadows also occurred with symmetrical distribution in the mid- and lower lung fields [28,61]. The pneumonic signs usually disappeared three to six months after effective etiological treatment. The pulmonary disease was mainly caused by egg granulomas. However, three reports confirmed that ectopic parasitism of *S. japonicum* adult worms in the lung was found at autopsy in four patients deceased due to acute schistosomiasis [62-64]. Taking into account that postmortem examination is quite rare and not easily done in patients deceased due to schistosomiasis, as well as from other diseases in China, adult *S. japonicum* parasitism in human lungs in acute schistosomiasis may not be infrequent.

In advanced schistosomiasis, diffuse pulmonary involvement with egg granulomas was common, i.e., in 18 (82%) of 22 cases at autopsy [65,66]. However, individuals with clinical pulmonary symptoms in chronic and advanced schistosomiasis were rarely diagnosed during their lifetimes. During a three-year period in the 1960s, in a big specialized hospital for schistosomiasis in Shanghai, only 10 cases of chronic cor pulmonale induced by schistosomiasis were observed, all in the advanced phase of the disease. During the same period, a total of 723 cases of advanced schistosomiasis were treated in the hospital. The rate of cardiopulmonary complication among cases with advanced schistosomiasis was 1.4%. None were observed in the chronic phase of the disease [65]. After the 1980s, along with the progress of the control program, pulmonary schistosomiasis, especially schistosomal cor pulmonale, has rarely been reported.

### Schistosomiasis and cancer

A number of epidemiological data have suggested that a close relationship exists between colorectal cancer and schistosomiasis in China. A nationwide retrospective survey of cancer in China, conducted between 1973 and 1975, showed that cancer of the large intestine was seen mainly in the provinces of Zhejiang, Jiangsu and Fujian, and the municipality of Shanghai**—**a distribution that coincides with areas that had a high prevalence and morbidity of schistosomiasis at the time [67]. By analyzing the mortality data of colorectal cancer and *S. japonicum* infection in 24 provinces in China covering a population of 850 million, a parallel correlation between the mortality rate due to colorectal cancer and schistosomiasis in endemic areas, especially in areas with high prevalence rates, was demonstrated by Chinese scientists in the 1980s [68]. In another study, the prevalence of *S. japonicum* was positively correlated with the mortality of colorectal cancer in five counties in the Jiangsu and Zhejiang provinces [69]. In the same publication, data at county level from the Haining County Cancer Registry of Zhejiang Province for 24 communes (now townships) showed that the incidence of colorectal cancer was significantly correlated with the prevalence of schistosomiasis [69]. The correlativity of geographical distribution between colorectal cancer and schistosomiasis was shown by a study in the Jiangxi Province in the 1970s [70]. The study also showed that the mortality due to large bowel cancer was significantly higher in residents in the areas endemic for schistosomiasis (4.85/100,000) than those in non-endemic areas (3.31/100,000). In a highly endemic area for schistosomiasis, Jiashan County, Zhejiang Province, the prevalence rate of cancer of the large intestine was 44.2/100,000, the highest in China [69]. A significant association between colorectal cancer and previous schistosome infection (odds ratio 3.3) was also reported from a matched, case–control study in three counties of the Sichuan Province [71]. Another case–control study on 252 cases of colorectal cancer in Kunshan County, Jiangsu Province compared with a matched control group showed a strong association (odds ratio 8.3) between rectal cancer and history of schistosomiasis [72]. In a retrospective review in 1984 on mortality due to all malignancies in seven *S. japonicum* endemic counties in the Jiangsu Province, it was shown that the prevalence rate of schistosomiasis and the mortality rate due to colorectal cancer increased in parallel [73], and persons with schistosomiasis had a significantly higher mortality rate due to colorectal cancer than those without [74]. In cases of intestinal cancer associated with schistosomiasis, the locality of the cancer was predominately seen in the rectum, as was also reported in Japan [75], followed by the sigmoid colon, and finally in the other part of the colon, while cancer in the small intestine was a rarity which coincides with relative distribution of *S. japonicum* eggs in the intestine of man [52,69,76-78]. In an analysis of 285 pathological specimens with colorectal cancer from surgical operations in a heavily endemic area for schistosomiasis, it was shown that in 220 cases in which the cancer was associated with schistosomiasis, cancer in the rectum and sigmoid colon accounted for 44% and 27%, respectively, while in the 65 cases in which the cancer was without schistosomiasis, the comparative figure in the rectum and sigmoid colon was 23% and 18%, respectively, a significant difference [78].

Schistosome infection may have a negative effect on the prognosis of colorectal cancer. In a report by the National Cooperative Group on Pathology and Prognosis of Colorectal Cancer [79], the five-year survival rate was 45.6% out of 430 cases complicated with schistosomiasis, which was significantly lower than in those without schistosomiasis (50.9% out of 2,717).

Although the data concerning schistosomiasis associated with colorectal cancer are very impressive, explanations for the association still remain speculative. Epithelial proliferation and polyp formation are associated with the malignant transition, and continuous epithelial proliferation adjacent to a chronic schistosomal ulcer seems to be precancerous [77]. Schistosome eggs as a causative agent for colorectal cancer may have the following mechanisms: physical effects, chemical effects, changes of the secretion and excretion of the biliary acid, and weakening of the cellular immunity of the host due to the infection [80]. Molecular etiology of rectal cancer in 22 advanced cases of schistosomiasis was studied compared with 22 paired cases of the cancer without the *S. japonicum* infection as a control using PCR-SSCP and DNA sequence analysis. In the group which had patients with schistosomiasis associated with rectal cancer, 13 mutations were found in 10 cases, whereas in the control group, only three revealed base pair substitutions at CpG dinucleotides (*P* < 0.05). This limited experimental observation suggested that the mutations in schistosomal rectal cancer may be the result of genotoxic agents produced endogenously through the course of schistosome infection [81].

Although the etiological association between *S. japonicum* and colorectal cancer has been accepted by most Chinese scientists, there are different opinions: the evidence in favor of the association between the two diseases may not be enough to be convincing [82-84]. Their arguments for the case and the data the latter used are comparatively weak. Further experimental studies on the relationship of the two diseases with molecular pathogenesis are needed.

The etiological relationship between hepatic schistosomiasis and primary liver cell cancer has been debated [85]. Based primarily on case–control studies and mortality analysis, previous and current schistosomal infection was found to be significantly associated with liver cancer both in China and outside China [71,75,86,87]. However, most epidemiological or clinical evidence indicates that hepatic schistosomiasis may not be a predisposing factor towards the development of liver cell cancer. In China, two large retrospective epidemiological surveys, one national and the other conducted in seven counties heavily endemic for schistosomiasis, showed no correlation between the *S. japonicum* infection and primary liver cancer [68,73]. No positive correlation between liver cancer and the *S. japonicum* infection was found in a case–control study with hospital patients in Japan [88]. As HBV and HCV infections are well-known hepatic carcinogens and individuals with *S. japonicum* infection usually have a higher association with HBV and HCV infection in China [89], the apparent increased frequency of liver cancer in patients with schistosomiasis is explained more accurately by the high frequency of associated HBV and, in rare cases, HCV infection and its sequela cirrhosis than by hepatoma [88-90]. However, based on animal experiments, a few authors have suggested that schistosomiasis may act as a co-carcinogen in some situations [1,91,92].

### Post-transmission schistosomiasis

Clinical problems associated with schistosomiasis will continue for a considerable period of time after transmission has been interrupted. In Puerto Rico, Japan, as well as other areas and countries, it has caused post-transmission disease [93]. Consequently, it has been proposed that clinical signs of the disease that persist after the disease has been cured in areas where transmission and reinfection are not a threat, be renamed to “Post-transmission Schistosomiasis” [94]. Along with the big achievements in schistosomiasis control in China, transmission interruption has been reached in large areas [36]. However, after the *S. japonicum* infection and transmission stop and no living worms remain in the hosts’ bodies, among a part of formerly infected and cured individuals, clinical disease can still develop. Therefore, disease control is still needed in the post-transmission period. A government policy concerning treatment and medical assistance to individuals with advanced schistosomiasis was implemented in 2003 in China. The policy covers patients not only in endemic areas, but also in areas where the transmission of schistosomiasis has been interrupted [95]. Since then, tens of thousands of advanced cases have received medical help. Recent longitudinal surveys in the areas of Huangshan City (Anhui Province), Jiaxing City (Zhejiang Province), and Changshu City (Jiangsu Province), which are formerly endemic areas for schistosomiasis where transmission had been interrupted for some 20 years, hundreds of newly-developed advanced cases were identified [96-99]. The identification of the so-called “newly discovered” or “newly developed” advanced schistosomiasis cases is based on 1) former schistosome infection assessed by fecal examination and treated with anti-schistosome chemicals, and with documented cure via repeated stool-examination follow-ups; 2) formerly diagnosed as chronic schistosomiasis cases without the signs of significant liver fibrosis and portal hypertension; 3) at the time of follow-ups, both fecal examination and serum immunological tests for schistosome infection were negative; and 4) at the time of follow-ups, the patients showed the symptoms and signs of ascites, splenomegaly with hypersplenism, upper gastrointestinal bleeding, hepatic failure, etc. Criteria for advanced schistosomiasis in China were reached through physical examinations, abdominal ultrasonography, liver and spleen function tests, and tests of serum fibrotic indices of the liver. The results of three investigations show that in those cases with advanced schistosomiasis, schistosome infection was stopped a long time ago, but the disease still insidiously developed. This is a problem China is facing for schistosomiasis control in the post-transmission period and so surveillance and suitable intervention are needed in areas where transmission is interrupted. In addition, further studies on the pathogenesis of post-transmission schistosomiasis are necessary.

### Morbidity in mammals

One outstanding presentation is that different from other *Schistosoma* species, many mammals can act as reservoir hosts of *S. japonicum*, which increases the complicity of the epidemiology and control of the disease both to humans and animal husbandry. Up until now, apart from humans, a total of 42 species in 28 genera under seven orders of mammals have been found to naturally harbor the *S. japonicum* infection in China [28,100,101]. Many investigations show that in most endemic areas, farm cattle and buffaloes are the most important sources of the *S. japonicum* infection and are thus responsible for more of the transmission, with humans, goats, sheep, and pigs of secondary importance, and wild mammals contributing little to the disease transmission [100].

Laboratory studies with artificial infection showed that the sensitivity to the *S. japonicum* infection in descending order among 13 species of the animal reservoir was as follows: mice, rabbits, dogs, goats, cattle, rhesus monkeys, guinea pigs, sheep, white rats (*Rattus norvegicus albus*), brown rats (*R. n. socer*), pigs, buffaloes, and horses [102]. Among them, the domestic animals of cattle, pigs, goats, sheep, and dogs were sensitive to the *S. japonicum* infection and the infection causes higher morbidity, while buffaloes and horses were not [102]. Many epidemiological surveys showed that human prevalence and morbidity of the *S. japonicum* infection is basically parallel with the domestic animal reservoirs [38-40,55,100]. Numerous farms raising cattle, sheep, goats, or pigs were closed as most, or all, of the domestic mammals died in the 1950s and 1960s in Hunan, Jiangxi, and Anhui provinces, mainly due to acute or heavy infection of the disease, which resulted in high economic loss in animal husbandry [31,34,103]. In marshland and lake regions of the endemic areas in China, animal reservoirs, mainly domestic mammals, are more important sources of the infection than humans in terms of the disease transmission. Relative egg contaminating indices on the marshlands accounted for 70–90% from cattle and buffaloes from the 1970s until the 1990s according to different field collections, with the index being much lower from humans [28,34,103]. One large-scale epidemiological and quantitative egg count survey showed that more than 90% of schistosome eggs in the flood plains around the Poyang Lake areas were excreted by bovine including yellow cattle and water buffaloes around 2001 [104]. Another epidemiological and quantitative egg count survey on an islet in the Yangtze River in the Anhui Province showed that bovine was responsible for the contamination with schistosome eggs in as high as 99.7% of the cases [105]. In the plain region and hilly region of the endemic areas, feces from bovine and wild rats may play a major role in the contamination of fields with schistosome eggs, while human feces play a less important role. Infection status of sources of *S. japonicum* in the marshland and lake region and hilly region in the Anhui Province has been studied recently via fecal collection and parasitological examination. The egg count showed that most of the eggs (97.9%) were from cattle and buffaloes, while only 2.1% were from humans. In a pilot survey conducted in the hilly region of Anhui, egg count showed that most (99.7%) of the eggs were from wild rats and dogs, and only 0.3% of the eggs were from humans [105]. Another survey from the mountainous region in the Yunnan Province in the 1990s showed that the egg contamination rate in the fields from cattle, humans, and other domestic animals was 61%, 33%, and 6%, respectively [41].

Data from three large-scale national sampling surveys on the epidemiology of schistosomiasis carried out in 1989, 1995, and 2004, respectively, showed that the prevalence of schistosomiasis was on the decline, although still relatively high (see Table 1). The prevalence of schistosome infection in cattle and buffaloes based on fecal examination in the three national surveys is provided with comparative human prevalence from the same surveys as references [38-40]. As a whole, the prevalence in bovine is usually higher than in humans. A nationwide survey conducted in China in 2011 on the infection of *S. japonicum* in domestic animals in seven endemic provinces showed a further significant reduction. The egg positive rate was 0.88% in bovine, 0.95% in ovine (sheep and goats), and 0.09% in other domestic animals including horses, donkeys, pigs, dogs, etc., as shown in Table 2[106]. In the five provinces, municipality, and autonomous region, in areas which had already reached the criteria of elimination of schistosomiasis between 1985 and 1997, no infected domestic animals were found from epidemiological surveillance conducted since then [34,100,101].

**Table 1:** **Prevalence of the *S. japonicum *infection in bovine in endemic areas from three nationwide sampling surveys with human prevalence as references **[38-40]

***Year***	***Animal***	***No. examined***	No. positive*	***Prevalence (%)***	***Reference: human prevalence in the same period (%)***
1989	Cattle	3,203	530	16.55	
	Buffalo	10,234	1,256	12.27	10.20*
	Total	13,437	1,786	13.29	
1995	Cattle	3,196	229	7.17	
	Buffalo	11,347	1,088	9.59	4.89*
	Total	14,543	1,317	9.06	
2004	Cattle	2,883	106	3.68	
	Buffalo	6,391	298	4.66	2.51**
	Total	9,274	404	4.36	

*Based on fecal examination for schistosome eggs.

**Correct positive rate based on serological screening and fecal examination.

**Table 2:** **Prevalence of *S. japonicum *infection in domestic animals in seven endemic provinces of China in 2011**

***Animal***	***Total no. of animals***	No. examined in the areas*	***No. positive***	***Positive rate (%)***	***Deduced positive no. of animals***
Bovine	1,298,966	784,679	6,897	0.88	8,433
Ovine	1,385,216	41,110	389	0.95	2,314
Other domestic animals**	162,366	30,005	26	0.09	147

*Based on fecal examination.

**Including horses, donkeys, pigs, dogs etc., and data from two provinces**—**Yunnan and Jiangsu**—**only.

## Conclusion

Schistosomiasis was once a big disaster in China and now it is under effective control. Historical data have shown that the morbidity was very high with considerable mortality. During the periods of those before 1949, in the early phase of the national control program in the 1950s up to 1970s, and later on, the prevalence, morbidity and mortality have been changed greatly. Clinical patterns and the images of the infected subjects have been changed substantially and in large areas now transmission is interrupted. However, China is still facing the problems in both the transmission and disease control: such as to further reduce the prevalence, morbidity and to interrupt the transmission in still endemic areas, to treat and rescue symptomatic patients (advanced cases) both in endemic areas and post-transmission areas, as well as to control and eliminate important sources of *S. japonicum* infection from domestic animal reservoirs (mainly cattle and buffaloes) are a long-term and hard task.

## Competing interests

The author declares that he has no competing interests.

## Supplementary Material

Additional file 1 Multilingual abstracts in the six official working languages of the United Nations. Click here for file
