# Supplementary material for: Assessment of morbidity due to Schistosoma japonicum infection in China
Source: Infect Dis Poverty. 2014 Feb 14;3:6. doi: 10.1186/2049-9957-3-6 (PMC3928580; doi:10.1186/2049-9957-3-6)
Supplement: Additional file 1 — Multilingual abstracts in the six official working languages of the United Nations. [file 2049-9957-3-6-S1.pdf]

Translation of the abstract into the six official working languages of the United Nations

تقييم لانتشار مرض البلهارسيا اليابانية بدولة الصين

مينج جانج شين

مخلص

يقدم البحث تقييم تاريخي لانتشار مرض البلهارسيا اليابانية بدولة الضين. نظرًا للظروف الاجتماعية والتي لم تسمح بتنفيذ برنامج احتوائي إلا في بداية الخمسينات من القرن الماضي، فقد كان انتشار المرض خطير وارتفعت معدلات الوفاة. طبقًا لأبحاث ودوريات سابقة، يمكننا أن نقول أن هذا المرض تسبب بوفاة الملايين ودمر العديد من العائلات والمدن. ومنذ بدايات عام 1950، بدأ تنفيذ برنامج احتواء محلي مكثف بالإضافة إلى برامج وقائية مما تسبب بالتبعية بالتحكم بالمرض. والآن، انخفض انتشار وسيطرة المرض بصورة ملحوظة. يوضح البحث انتشار المراحل الثلاثة للمرض. يظهر الانتشار الشديد بالمرحلتين الحادة والمتقدمة من المرض. كما يشير البحث إلى الأربعة أشكال المتقدمة من المرض وهي: الاستسقاء وتضخم الطحال والقزامة وتكاثر بطانة القولون مع توضيح الصفات السريرية ونسب كل منهم بكل فترة من الفترات المختلفة من برنامج الاحتواء المحلي. كما نناقش البلهارسيا والعلاقة بين البلهارسيا اليابانية وسرطان القولون. كما نناقش بصورة مختصرة مرحلة ما قبل نقل المرض (وهو ما يحدث عند توقف مرحلة التخلص والعدوى والنقل ولكن مازالت تتطور) . وما يجعل المشكلة أكثر تعقيدًا بالصين هو الثدييات المضيفة ويقدم البحث التقدم بالتحكم بالحيوانات الحافظة للمرض

Translated from English version into Arabic by Laila Mostafa, through

TRANSLATORS WITHOUT BORDERS

中国的日本血吸虫病的危害性

陈名刚

摘要

本文就中国的日本血吸虫病的危害性进行历史评述。在上世纪 50 年代以前，限于社会经济条件，没有全国血吸虫病防治规划，血吸虫病危害严重，死亡率高。根据一些调查和文献记载，血吸虫病导致数以百万计的人员病死、无数家庭被毁、不少村庄消亡。1950 年代起，全国大力开展血防工作，使疾病得到有力控制，危害性大为减轻。中国血吸虫病分为急性、慢性和晚期 3 期，而以急性和晚期的危害较重。根据不同的临床表现，中国把晚期血吸虫病分为 4 个类型：腹水、巨脾、侏儒和结肠增殖型。在全国防治规划实施的不同阶段，它们所占比例有很大变化。本文对异位血吸虫病和血吸虫感染与大肠癌的关系进行了论述。在血吸虫病得到消除的地区，存在传播阻断后血吸虫病问题。哺乳类动物的日本血吸虫储存宿主问题使中国的血吸虫病流行病学复杂化，防治工作更为艰巨。对动物血吸虫病的防治进展进行了简要陈述。

Translated from English version into Chinese by Chen Ming-gang

Évaluation de la morbidité due à l'infection au *Schistosoma japonicum* in China

Ming-gang Chen

Résumé

Cet article présente une évaluation historique de la morbidité due à l'infection à Schistosoma japonicum en Chine. En raison de la situation socioéconomique qui n'a pas permis de mettre un programme de contrôle en œuvre avant le début des années 1950, avant cette date, la morbidité était grave et la mortalité était élevée. Sur la base de quelques enquêtes et articles publiés, on peut dire que la maladie a causé des millions de morts et détruit de nombreux villages et familles. Depuis les années 1950, un programme national de contrôle existe, un travail intensif de contrôle et de prévention a été effectué, et par conséquent la maladie est maîtrisée. À l'heure actuelle, la prévalence et la morbidité de la maladie ont diminué sensiblement. La morbidité des trois phases de la maladie est décrite dans ce document. Une morbidité comparativement plus élevée est observée dans les phases aiguës et avancées de la maladie. Les quatre principales formes de schistosomiase avancées, c'est à dire, ascites, mégalosplénie, nanisme et une prolifération tumoroïde colique sont décrites avec leurs présentations cliniques caractéristiques ; leurs proportions relatives ont varié au cours des diverses périodes du programme de contrôle national. La schistosomiase ectopique et la relation entre l'infection à S. japonicum et le cancer colorectal sont également discutées. La schistosomiase post-transmission est brièvement discutée (celle qui peut arriver et continuer à se développer alors que la maladie a atteint les critères d'élimination et que l'infection et la transmission ont cessé). Le problème des mammifères réservoirs de S. japonicum rend l'épidémiologie et le contrôle de la schistosomiase en Chine encore plus compliqué et ardu ; les progrès de contrôle des réservoirs animaux sont brièvement présentés.

Translated from English version into French by Michele Lemaire, through

TRANSLATORS
WITHOUT BORDERS

Оценка заболеваемости, вызванной возбудителем *Schistosoma japonicum* в Китае

Минган Чэн

Аннотация
В работе представлена историческая оценка заболеваемости, вызванной возбудителем *Schistosoma japonicum* в Китае. В связи с социально-экономическими обстоятельствами уровень заболеваемости и смертности от этой инфекции в Китае оставался высоким вплоть до начала 1950-х годов, когда была внедрена программа контроля. На основании нескольких исследований и опубликованных работ делается вывод о том, что данное заболевание вызвало миллионы смертей и разрушило множество семей и поселений. Начиная с 1950-х годов, после запуска национальной программы контроля принимаются меры по контролю заболевания и проводится профилактическая работа. В настоящее время распространенность японского шистосомоза и уровень заболеваемости значительно снизились. В работе приводятся данные по распространенности трех стадий болезни. Сравнительно большее количество случаев заболевания было выявлено на острой и поздней стадиях. В работе описаны четыре формы шистосомоза на поздней стадии (асцит, спленомегалия, карликовость и разрастание опухолевидных образований в кишечнике) и их типичные клинические проявления; их пропорции варьируются в разные периоды реализации национальной программы контроля. Кроме того, обсуждается эктопический шистосомоз и связь между японским шистосомозом и колоректальным раком. Также затрагивается вопрос о шистосомозе, возникающем после прекращения эпидемического распространения и передачи возбудителя. Проблема переноса возбудителей инфекции млекопитающими еще больше осложняет эпидемиологию и контроль шистосомоза в Китае; в работе кратко рассматриваются достижения в вопросе контроля за животными-носителями возбудителя инфекции.

Translated from English version into Russian by Sophia Shishatskaya, through

TRANSLATORS
WITHOUT BORDERS

Evaluación de la morbilidad debida a infección por *Schistosoma japonicum* en China

Ming-gang Chen

Abstracto

Este artículo presenta una evaluación histórica de la morbilidad debida a infección por *Schistosoma japonicum* en China. Debido a la situación socioeconómica, que no permitió la implantación de un programa de control hasta principios de la década de 1950, hasta entonces la morbilidad era grave y la mortalidad alta. Según algunas investigaciones y artículos publicados, puede decirse que la enfermedad causaba millones de muertes, y destruía numerosas familias y pueblos. Desde la década de 1950, ha existido un programa nacional de control, se ha llevado a cabo un trabajo intensivo de control y prevención, y en consecuencia la enfermedad está siendo controlada. Actualmente, han descendido substancialmente tanto la morbilidad asociada a la enfermedad como su prevalencia. En este artículo se presenta la morbilidad asociada a las tres fases de la enfermedad. Se observa una morbilidad comparativamente mayor en las fases aguda y avanzada de la enfermedad. Se exponen las cuatro formas principales de esquistosomiasis avanzada, es decir ascitis, esplenomegalia, enanismo, y proliferación tumoroide en el colon, junto con su presentación clínica característica; su proporción es diferente durante varios periodos del programa de control nacional. Se habla asimismo de la esquistosomiasis ectópica y la relación entre la infección por *S. japonicum* y el cáncer colorrectal. Se trata brevemente la esquistosomiasis posterior a la transmisión (que puede ocurrir incluso si la enfermedad alcanza los criterios de eliminación y la infección y su transmisión se han detenido, pero aún así se desarrolla). El problema de los mamíferos que actúan como huéspedes reservorios de *S. japonicum* hace que la epidemiología y control de la esquistosomiasis en China sea aún más complicada y ardua, y se presenta brevemente el progreso realizado en el control de reservorios animales.

Translated from English version into Spanish by Cristina Novo, through

TRANSLATORS
WITHOUT BORDERS
